# Comparison of Relative Survival and Cause-Specific Survival in Men With Prostate Cancer According to Age and Risk Category: A Nationwide, Population-Based Study

**DOI:** 10.1093/aje/kwab146

**Published:** 2021-05-19

**Authors:** Andri Wilberg Orrason, Hans Garmo, Johan Styrke, Paul W Dickman, Pär Stattin

**Keywords:** cause-specific survival, net survival prostate cancer, prognosis, relative survival, survival

## Abstract

Net survival, estimated in a relative survival (RS) or cause-specific survival (CSS) framework, is a key measure of the effectiveness of cancer management. We compared RS and CSS in men with prostate cancer (PCa) according to age and risk category, using Prostate Cancer data Base Sweden, including 168,793 men younger than age 90 years, diagnosed 1998–2016 with PCa. RS and CSS were compared according to age and risk category based on TNM (tumor, nodes, and metastases) stage, Gleason score, and prostate-specific antigen level. Each framework requires assumptions that are unlikely to be appropriate for PCa. Ten-year RS was substantially higher than CSS in men aged 80–89 with low-risk PCa: 125% (95% confidence interval: 113, 138) versus 85% (95% confidence interval: 82, 88). In contrast, RS and CSS were similar for men under age 70 and for all men with regional or distant metastases. Both RS and CSS produce biased estimates of net survival for men with low- and intermediate-risk PCa, in particular for men over 80. Due to biases, net survival is overestimated in analysis of RS but underestimated in analysis of CSS. These results highlight the importance of evaluating the underlying assumptions for each method, because the “true” net survival is expected to lie between the limits of RS and CSS.

## Abbreviations

CI: confidence interval
CSS: cause-specific survival
NPCR: National Prostate Cancer Register
PCa: prostate cancer
PSA: prostate-specific antigen
RS: relative survival

Prostate cancer (PCa) is a common cancer in high-resource countries. The incidence of PCa has risen during the last 2 decades, mostly due to an increased detection of low- and intermediate-risk disease due to testing of serum levels of prostate-specific antigen (PSA) and ensuing ultrasound guided biopsies of the prostate (1, 2). Concomitantly, there have been improvements in the workup and treatment of PCa, with large increases in the use of radical treatments, and in many countries, there has been a decline in PCa mortality (2–4).

**Figure 1:**
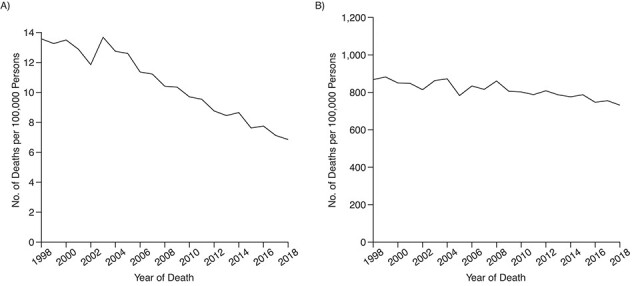
Age-standardized prostate cancer mortality for men younger than age 80 years (A) and men aged 80 or older (B), Sweden, 1998–2018 (5).

In Sweden, the incidence of PCa, age-adjusted to the world population, increased over 1998–2017 from 74 to 94 per 100,000 men, and PCa mortality decreased from 22 to 14/100,000 (5). In men under age 80, the decrease in PCa mortality was around 50%, from 14 to 7 per 100,000, whereas in men age 80 or older, the mortality declined only 13%, from 868 to 755 per 100,000 (Figure 1).

Mortality is considered to be a robust measure of progress in cancer control because it is not substantially affected by overdiagnosis. However, the total change in PCa mortality has been quite modest, despite large efforts devoted to early detection and early treatment of PCa, due to the fact that a large proportion of men who die of PCa are over age 80, for whom PCa might be overreported as a cause of death (6, 7).

Mortality captures the combined effects of primary prevention (which affects incidence), secondary prevention, and treatment. It is also desirable to measure the effectiveness of cancer management (i.e., diagnosis and treatment) using a measure that is not affected by changes in incidence. Net survival is a measure of patient survival that is independent of changes in incidence and independent of background (noncancer) mortality. Net survival is interpreted as the probability of survival in the hypothetical situation in which death can occur only due the disease under study. It can be estimated using 2 different frameworks; relative survival (RS), based on comparison with a disease-free background population, or cause-specific survival (CSS), based on classification of underlying cause of death.

In population-based studies of cancer survival, the relative survival framework is considered most suitable for estimating net survival (6, 8). RS requires the assumption that factors affecting survival, not captured by life tables (e.g., comorbidity and socioeconomic status), are similar in the study population and in the background population, whereas CSS requires correct classification of cause of death. However, in older men with PCa, these assumptions might not hold true due to incomparability with respect to comorbidity in the general population and a higher risk of a misclassification of cause of death.

Multiple registry-based studies have compared these 2 measures of survival for men with PCa, and RS has consistently been higher than CSS (6, 7, 9–11). However, these studies did not include data on PCa risk category, and some of the studies used age groups with a cutoff of age 65 years, which is too low for meaningful analyses (9).

The aim of this study was to compare RS and CSS according to age and risk category in men with PCa in a large population-based cohort with comprehensive data on cancer characteristics, treatment, and cause of death. A unique feature of our study is that we have detailed data on comorbidity of both men with PCa and matched comparators, which can potentially be used to correct the well-known bias in RS.

## METHODS

### Registers

The National Prostate Cancer Register (NPCR) of Sweden holds information on 98% of all incident PCa cases since 1998, as compared with the Swedish Cancer Registry, to which reporting is mandated by law. NPCR contains detailed data on cancer characteristics, diagnostic workup, and primary treatment. Using the unique Swedish personal identity number, NPCR has been linked to a number of other nationwide health-care registers and demographic databases—including the Patient Registry, the Prescribed Drug Registry, the Cause of Death Registry, and the longitudinal integration database for health insurance and labor market studies—to construct the Prostate Cancer data Base Sweden (12). This research database also includes 5 matched comparators for each man with PCa. Comparators were randomly selected, using the Total Population Register maintained by Statistics Sweden, from all men in the population who were free of PCa at end of the year of diagnosis of the index case, resided in the same county as the case, and were born in the same year.

### Study population

We used Prostate Cancer data Base Sweden, version 4.1, and categorized the study population by age and risk category at date of diagnosis. Age groups were defined, in years: <70, 70–79, and 80–89; men 90 years or older at diagnosis were excluded due to short life expectancy. Risk category was defined according to a modified National Comprehensive Cancer Network categorization: 1) low-risk: clinical (tumor, nodes, metastases) stage T1–T2, Gleason score 6, and PSA <10 ng/mL; 2) intermediate-risk: T1–T2 and Gleason score 7, or PSA 10–19.9 ng/mL; 3) high-risk or locally advanced: T3, Gleason score 8 or above, or PSA 20–49.9 ng/mL; 4) regional: T4, N1, or PSA 50–99.9 ng/mL; and 5) distant metastases: M1 or PSA ≥100 ng/mL.

Follow-up started at the date of diagnosis and ended at December 31, 2017, date of emigration, or date of death, whichever occurred first.

### Statistical analysis

RS was estimated with the Ederer II method (13). Expected survival was calculated using survival probabilities from life tables generated from the Swedish population, matched by age and year of the study population (14). CSS was estimated with the Kaplan-Meier method (15). The so-called “complete” follow-up approach was applied in both methods as described by Brenner and Rachet (16). Estimates from both methods were plotted and stratified by age and risk category.

In order to maximize the comparability between the study population and the general population we repeated the RS analysis using PCa-free comparators in Prostate Cancer data Base Sweden instead of data from general life tables. Comparators were matched, by design, to the study population on age and calendar year at date of PCa diagnosis, as well as for factors not captured by general life tables (i.e., comorbidity, educational level, and marital/partnership status). Comorbidity at date of diagnosis was classified by use of the Charlson Comorbidity Index, which is a weighted sum of a number of *International Classification of Diseases* codes for discharge diagnoses excluding PCa in the Patient Registry (17). Comorbidity was classified into 4 levels: 0, 1, 2 or ≥3. In addition, a novel Drug Comorbidity Index was used that predicts an individual’s risk of death from any cause based on drug prescriptions the year prior to diagnosis (18). Matching of comparators was performed using cases diagnosed during 2007–2016. (The Drug Comorbidity Index was available from July 2005. when the Prescribed Drug Registry was initiated.) Matching was based on exact values of the cases’ covariates, except Drug Comorbidity Index, which was allowed to deviate 0.5 points. After matching, RS was estimated by dividing the ratio of overall survival of cases with overall survival of comparators, whereby the follow-up of comparators was censored according to the Ederer II method (i.e., at the time of death or censoring of their matched case). Furthermore, matching on age and year of diagnosis only was repeated using comparators 1 year younger than their matched cases, and then with comparators 2 years younger than their cases.

The analysis was performed using R, version 3.4.2 (Foundation for Statistical Computing, Vienna, Austria), using packages survival, relsurv, and Matching. The Research Ethics Board in Uppsala approved the study.

## RESULTS

A total of 168,793 men diagnosed with PCa were registered in NPCR during the study period (Table 1). At date of PCa diagnosis, men older than 80 years, compared with men under 70, more often had advanced local tumor stage (T3–T4) (44% vs. 14%), higher PSA levels (median, 29 vs. 8 ng/mL), more often had Gleason score 8 or 9–10 (33% vs. 13%) and more often had distant metastases (14% vs. 6%). Furthermore, men over 80 almost never received primary radical treatment (1%), whereas 58% of men under 70 received primary radical treatment.

**Table 1:** Baseline Characteristics for Men with Prostate Cancer in Prostate Cancer data Base Sweden, Version 4.1, According to Age at Diagnosis Between 1998–2016

**Characteristic**	**Age, years**							
	**<70 (*n* = 83,533)**		**70–79 (*n* = 58,697)**		**≥80 (*n* = 26,555)**		**Total (*n* = 168,793)**	
	**No.**	**%**	**No.**	**%**	**No.**	**%**	**No.**	**%**
Age, years^a^	64 (60, 67)		74 (72, 77)		83 (81, 85)		70 (64, 76)	
Clinical tumor stage								
T1	47,491	57	22,384	38	6,000	23	75,875	45
T2	23,018	28	19,578	33	8,660	33	51,257	30
T3	9,944	12	12,927	22	9,069	34	31,943	19
T4	1,548	2	2,204	4	1,943	7	5,697	3
Missing	1,532	2	1,604	3	883	3	4,021	2
Node stage								
N0	14,324	17	5,725	10	1,167	4	21,216	13
N1	1,866	2	1,026	2	322	1	3,215	2
Nx	67,343	81	51,946	88	25,066	94	144,362	86
Metastasis stage								
M0^b^	23,042	28	16,893	29	4,642	17	44,577	26
M1	4,681	6	5,805	10	3,739	14	14,227	8
Mx	55,810	67	35,999	61	18,174	68	109,989	65
Serum PSA ng/mL^a^	8 (5, 14)		13 (7, 34)		29 (13, 82)		10 (6, 27)	
<10	52,326	63	22,199	38	4,366	16	78,891	47
10–49.9	22,623	27	23,853	41	12,033	45	58,509	35
50–99.9	2,877	3	4,322	7	3,574	13	10,776	6
≥100	4,479	5	6,584	11	5,498	21	16,566	10
Missing	1,228	1	1,739	3	1,084	4	4,051	2
Gleason score								
≤6	42,909	51	19,723	34	5,153	19	67,785	40
7	26,381	31	19,763	34	4,419	19	53,961	32
8	5,700	7	6,489	11	4,124	16	16,313	10
9–10	4,741	6	6,266	11	4,462	17	15,471	9
WHO grade^c^	3,052	4	5,550	9	3,905	15	12,507	7
Missing	750	1	906	2	1,095	4	2,756	2
Primary treatment								
Radiotherapy	14,843	18	8,056	14	162	1	23,061	14
Radical prostatectomy	33,761	40	5,023	9	65	0	38,849	23
ADT	10,769	13	23,641	40	18,389	69	52,807	31
AS or WW	20,583	25	19,613	33	7,041	27	47,237	28
Missing	3,577	4	2,364	4	898	3	6,839	4
Charlson Comorbidity Index								
0	70,590	85	40,298	69	14,571	55	125,462	74
1	7,756	9	9,655	16	5,770	22	23,184	14
2	3,150	4	4,914	8	3,190	12	11,256	7
≥3	2,037	2	3,830	7	3,024	11	8,891	5
Drug Comorbidity Index^a^^,^^d^	0.6 (0, 1.5)		1.2 (0.4, 2.4)		2.0 (1.0, 3.4)		0.9 (0.3, 2.1)	
Educational level^e^								
Low	24,320	29	26,559	45	14,471	54	65,353	39
Middle	34,776	42	20,535	35	7,880	30	63,192	37
High	24,007	29	10,965	19	3,524	13	38,497	23
Missing	430	1	638	1	680	3	1,751	1
Civil status								
Married/partnership	56,437	68	40,618	69	16,065	60	113,123	67
Unmarried	27,050	32	18,062	31	10,488	39	55,605	33
Missing	46	0	17	0	2	0	65	0
Risk category								
Low risk^f^	32,010	38	10,465	18	1,613	6	44,088	26
Intermediate risk^g^	25,926	31	16,278	28	4,140	16	46,344	27
High risk^h^	13,247	16	16,391	28	9,544	36	39,182	23
Regional metastases^i^	3,835	5	4,602	8	3,470	13	11,910	7
Distant metastases^j^	6,714	8	9,262	16	7,096	27	23,077	14
Missing	1,801	2	1,699	3	692	3	4,192	2

Abbreviations: ADT, androgen deprivation therapy; AS, active surveillance; PSA, prostate-specific antigen; WHO, World Health Organization; WW, watchful waiting.

^a^ Values are expressed as median (interquartile range).

^b^ M0: No signs of distant metastases on bone imaging.

^c^ Only WHO grading available.

^d^ Predicts individual’s overall mortality risk based on drug prescription history the previous year.

^e^ Low: no more than 9 years (elementary school); intermediate: 9–12 years (secondary school); high: more than 12 years (college/university).

^f^ Clinical stage (tumor, node, metastasis) T1–T2, Gleason score ≤6, and PSA <10 ng/mL.

^g^ Clinical stage T1–T2 and Gleason score 7, or PSA 10–19.9 ng/mL.

^h^ Clinical stage T3, Gleason score 9–10, or PSA 20–49.9 ng/mL.

^i^ Clinical stage T4, N1, or PSA 50–99.9 ng/mL.

^j^ M1 or PSA ≥100 ng/mL.

### Relative versus cause-specific survival

**Figure 2:**
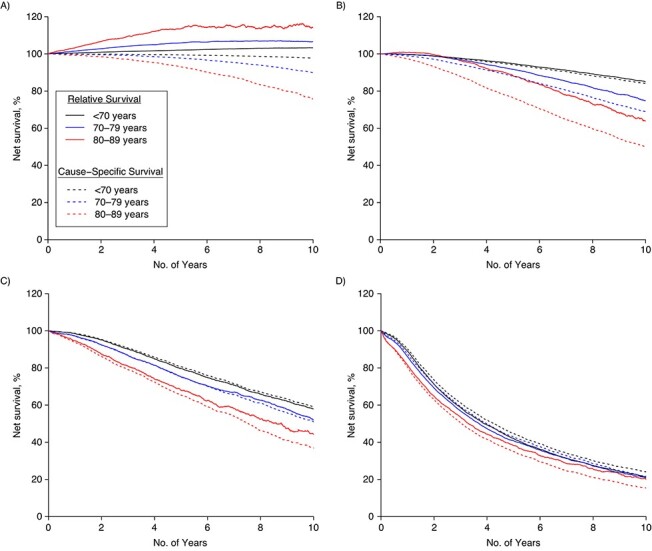
Relative and cause-specific survival according to age and risk category, among Swedish men after diagnosis of prostate cancer in 1998–2016. A) Low and intermediate risk; B) high risk; C) regional metastases; D) distant metastases.

The RS and CSS for the entire cohort at 5 years after diagnosis were 90% and 87%, respectively, and 84% and 77%, respectively, at 10 years. Men older than 80 with low-risk PCa at diagnosis had a particularly large difference between 5-year RS and CSS, 116% (95% confidence interval (CI): 112, 121) versus 96% (95% CI: 95, 97), and these differences were also seen in men with intermediate-risk PCa and to a lesser extent in men with high-risk PCa (Table 2, Figure 2).

**Table 2:** Relative Survival and Cause-Specific Survival at 5 and 10 Years After Diagnosis of Prostate Cancer, According to Age Group and Risk Category, Among Men in the Prostate Cancer Data Base Sweden, Diagnosed Between 1998–2016

**Age Group and Risk Category**	**5-Year Survival**				**10-Year Survival**			
	**RS**	**95% CI**	**CSS**	**95% CI**	**RS**	**95% CI**	**CSS**	**95% CI**
Low risk^a^								
<70 years	102.6	102.4, 102.8	99.8	99.7, 99.8	105.1	104.6, 105.6	98.9	98.8, 99.1
70–79 years	108.1	107.3, 108.9	98.9	98.7, 99.1	114.5	112.5, 116.5	94.2	93.6, 94.9
80–89 years	116.2	111.8, 120.8	95.8	94.6, 96.9	125.0	113.4, 137.7	85.0	82.1, 88.1
Intermediate risk^b^								
<70 years	101.3	101.0, 101.6	99.1	99.0, 99.2	100.8	100.1, 101.5	96.1	95.8, 96.4
70–79 years	104.4	103.7, 105.2	97.0	96.7, 97.3	100.8	99.0, 102.7	86.9	86.1, 87.7
80–89 years	112.3	109.4, 115.3	92.0	91.0, 93.0	108.9	101.1, 117.2	71.5	69.0, 74.2
High risk^c^								
<70 years	94.8	94.2, 95.4	94.2	93.8, 94.7	84.9	83.8, 86.1	84.0	83.2, 84.8
70–79 years	91.7	90.7, 92.6	88.0	87.5, 88.6	75.0	73.3, 76.7	69.0	68.0, 70.0
80–89 years	88.3	86.3, 90.4	76.4	75.3, 77.4	63.9	59.7, 68.5	50.1	48.2, 52.0
Regional metastases^d^								
<70 years	79.6	78.0, 81.2	80.6	79.3, 82.0	57.9	55.7, 60.2	58.9	57.0, 60.9
70–79 years	75.2	73.3, 77.1	75.3	73.9, 76.7	52.0	49.2, 55.0	51.0	49.0, 53.0
80–89 years	68.0	64.8, 71.4	65.4	63.4, 67.4	44.2	38.4, 50.8	36.8	33.8, 40.1
Distant metastases^e^								
<70 years	42.0	40.7, 43.4	44.7	43.4, 46.1	21.4	20.1, 22.8	24.1	22.8, 25.6
70–79 years	40.8	39.5, 42.1	42.9	41.7, 44.1	20.8	19.4, 22.4	21.7	20.5, 23.0
80–89 years	38.3	36.5, 40.3	35.1	33.7, 36.5	19.9	17.1, 23.2	15.4	13.9, 17.1

Abbreviation: CI, confidence interval, CSS, cause-specific survival; RS, relative survival.

^a^ Clinical (tumor, node, metastasis) stage T1–T2, Gleason score ≤6, and PSA <10 ng/mL.

^b^ Clinical stage T1–T2 and Gleason score 7, or PSA 10–19.9 ng/mL.

^c^ Clinical stage T3, Gleason score 9–10, or PSA 20–49.9 ng/mL.

^d^ Clinical stage T4, N1, or PSA 50–99.9 ng/mL.

^e^ M1 or PSA ≥100 ng/mL.

In contrast, 5-year RS and CSS were similar for men under 70 with low-risk PCa, 103% (95% CI: 102, 103) versus 100% (95% CI: 100, 100), as well as for all men with regional or distant metastases, with a difference of 3% or less between RS and CSS estimates.

The difference between RS and CSS for men over 80 continued to increase at 10 years after diagnosis: 125% (95% CI: 113, 138) versus 85% (95% CI: 82, 88) for low-risk PCa and 109% (95% CI: 101, 117) versus 72% (95% CI: 69, 74) for intermediate-risk PCa. The difference in RS and CSS did not substantially increase at 10 years for men over 80 with high-risk disease or for regional or distant metastases (Table 2**)**.

Ten-year RS in men under 70 with low-risk PCa was 105% (95% CI 105, 106) compared with a 10-year CSS of 99% (95% CI: 99, 99), whereas in men under 70 with regional or distant metastases RS and CSS were comparable, with a difference of 3% or less (Table 2**)**.

### Relative survival in men aged 80–89 years using matched comparators

**Figure 3:**
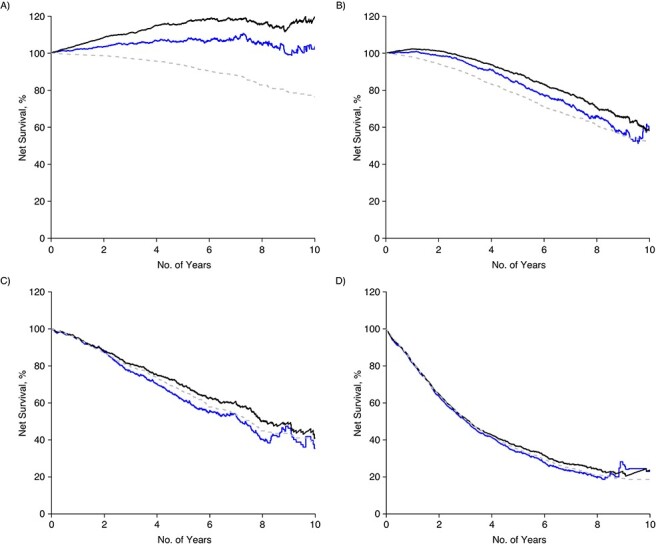
Net survival according to risk category, among Swedish men aged 80–89 years, after diagnosis with prostate cancer in 1998–2016. A) Low and intermediate risk; B) high risk; C) regional metastases; D) distant metastases. Relative survival measured with comparators matched by age and calendar year (solid black line) and comparators matched by age, calendar year, comorbidity, educational level, and marital/partnered status (solid blue line). Cause-specific survival shown in gray.

After matching on comorbidity (based on Charlson Comorbidity Index and Drug Comorbidity Index), educational level, and marital/partnership status, the RS decreased from 116% to 106% at 5 years and from 120% to 104% at 10 years for men with low- or intermediate-risk PCa (Figure 3). The decline in RS was less pronounced in men with high-risk PCa and men with regional metastases. However, the RS of men with distant metastases remained essentially unchanged after additional matching.

**Figure 4:**
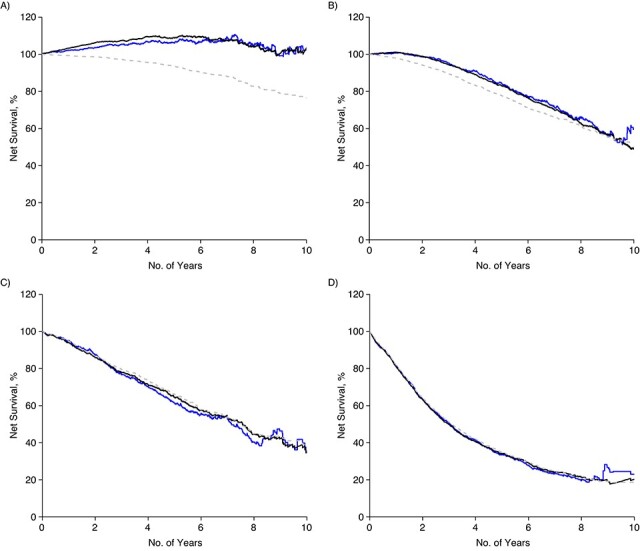
Net survival according to risk category, among Swedish men aged 80–89 years, after diagnosis with prostate cancer in 1998–2016. A) Low and intermediate risk; B) high risk; C) regional metastases; D) distant metastases. Relative survival measured with comparators matched by age (comparators 1 year younger) and calendar year (solid black line) and comparators matched by age, calendar year, comorbidity, educational level, and marital/partnered status (solid blue line). Cause-specific survival shown in gray.

**Figure 5:**
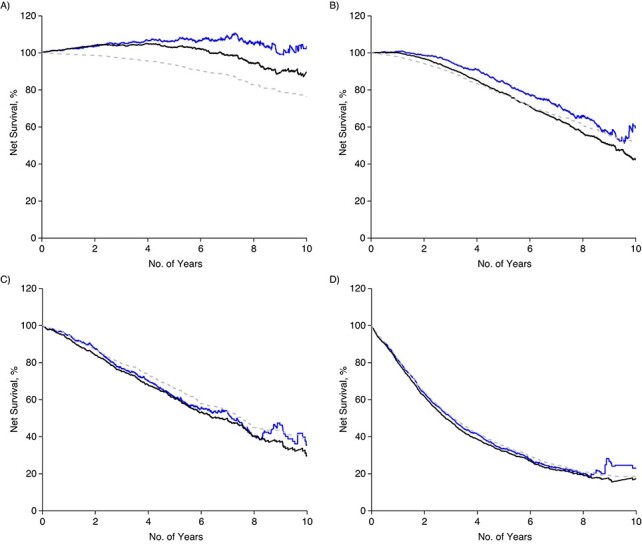
Net survival according to risk category, among Swedish men aged 80–89 years, after diagnosis with prostate cancer in 1998–2016: A) Low and intermediate risk; B) high risk; C) regional metastases; D) distant metastases. Relative survival measured with comparators matched by age (comparators 2 years younger) and calendar year (solid black line) and comparators matched by age, calendar year, comorbidity, educational level, and marital/partnered status (solid blue line). Cause-specific survival shown in gray.

The RS of men aged 80–89 years with low- or intermediate-risk PCa and those with high-risk PCa matched to comparators in Prostate Cancer data Base Sweden only by calendar year and age resulted in RS similar to that estimated by the life table method. Matching to comparators 1 year younger than their case resulted in RS similar to that from additionally matching by same age, comorbidity, educational level, and marital/partnership status (Figure 4). Matching to comparators 2 years younger led to further reduction in RS compared with the fully matched analysis, although still higher than CSS in men with low- or intermediate-risk PCa (Figure 5). Matching on comparators 1 or 2 years younger did not affect the RS of men with regional to distant metastasis at diagnosis.

## DISCUSSION

In this population-based registry study, RS was substantially higher than CSS for men with low- and intermediate-risk PCa, in particular for men aged 80–89 years. For these men, RS was above 100%, suggesting that older men diagnosed with PCa are healthier than their age-matched comparators in the background population. The low CSS suggests misclassification bias resulting in an inflated proportion of very old men who were adjudicated PCa as cause of death. The “true” net survival lies between RS and CSS and is a useful metric of long-term outcome of cancer care. Combining results from these 2 frameworks of net survival is helpful to interpret changes in cancer mortality.

The NPCR of Sweden has a 98% capture rate compared with the Swedish Cancer Registry, to which reporting is mandated by law. Thus, our study included virtually all men diagnosed with prostate cancer in a well-defined population during a 19-year period with a recent end of follow-up. In addition, completeness and validity of data in NPCR have been assessed and found to be high, and we also had access to data of high quality from other national health-care registers and demographic databases (19–21). To our knowledge, this is the first study to compare 5- and 10-year RS and CSS in men with PCa according to age and risk categorization based on tumor stage, PSA levels, and Gleason score. Access to data on comorbidity and socioeconomic data in age-matched comparators allowed us to further investigate limitations in RS due to incomparability when using general population life tables.

Our study has some limitations. Despite the large study population, there were relatively few men aged 80–89 years who were alive 10 years after diagnosis. Matching analysis using a Drug Comorbidity Index could be performed only for men diagnosed during 2007–2016, because the Prescribed Drug Registry, on which this index is based, started in July 2005. Although this limits the number of cases in the study, we argue that the restriction in study period is an advantage because these results are more representative for men currently diagnosed with PCa than if we had used the entire NPCR with men diagnosed from 1998.

Our observations are in accordance with other studies on RS and CSS in men with PCa, which have all shown higher RS compared with CSS—and the difference has been higher in men with less-advanced cancer and in older men (6, 7, 9–11). In a recent study by Forjaz de Lacerda et al. (6), using data from the Surveillance, Epidemiology, and End Result (SEER) database, cancers commonly detected by screening had a slightly higher RS than CSS, whereas cancers with distinct risk factors (e.g., cervical cancer) had a higher CSS than RS. However, the SEER data only included men over age 65 years, and follow-up was only 5 years. In a population-based study from Norway, RS was markedly higher than CSS in men over age 85, and this difference increased with time from diagnosis (7).

The Ederer II method was used to estimate RS in our study. Newer methods are available, such as the Pohar-Perme estimator, which is considered the only truly unbiased estimator of net survival in that it adjusts for dependent censoring with inverse probability weighting (22). However, this method is susceptible to high variability in estimates of survival at long follow-up times, whereas the bias in the Ederer II method due to dependent censoring is negligible when the study population is stratified by age (23).

General population life tables include men with PCa when expected survival probabilities are estimated. For cancer at most sites other than the prostate this is not an important issue; however, PCa is a common disease among older men and accounts for 5%–7% of all deaths in Swedish men older than 80 years and will hence decrease survival in the general population (5, 24). Our analysis based on the comparison with PCa-free comparators was not affected by this bias.

In men with low- or intermediate-risk PCa, RS was substantially higher than CSS, in particular among men over age 80. The reasons for this discrepancy are likely related to biases. In the relative survival framework, net survival is overestimated in older men with low-risk PCa due to a “healthy screening effect.” In our analysis, we were able to limit but not eliminate this bias by using comparators matched by age, comorbidity, educational level, and marital/partnership status. However, despite this matching, RS was still above 100% at 5 years after diagnosis for men with low- to intermediate-risk PCa. Unmeasured factors related to good health explain the higher survival rate of these men compared with the general population. Men diagnosed with PCa over age 80 are likely to be a selection of healthy men with a long life expectancy, because in order for a prostate biopsy to be considered for a man over 80, he must be fit. Furthermore, RS might be substantially affected by public health awareness and educational level, which could differ between populations.

The matching on age and calendar year was repeated using younger comparators to estimate the absolute difference in biological age of men with PCa compared with the general population of the same chronological age. Our results indicated that men aged 80–89 with low- or intermediate-risk PCa were biologically approximately 1 year younger than their chronological age. This difference might in fact be even larger; RS was still above 100% after this adjustment, which only accounted for differences in comorbidity, educational level, and marital/partnership status.

Cause-specific survival relies on correct assessment of the cause of death, and this is likely increasingly difficult with age because very old men often have high comorbidity. According to a study by Fall et al. (25), the validity of the Cause of Death Registry in Sweden was high for PCa death. Nevertheless, in that study, men over age 75 had a 5% higher risk of PCa death in the Cause of Death Registry compared with adjudication of PCa death after review of medical records, and this is possibly even higher in very old men with low-risk PCa and multiple comorbidities, who are more likely to die from competing causes than of their PCa.

In men with regional or distant metastatic PCa at diagnosis, RS and CSS resulted in very similar estimates in all age groups. The risk of misclassifying cause of death in men with advanced disease at diagnosis is small because most of these men die from PCa. Furthermore, men with metastatic PCa are likely more similar to the background population in terms of general health than men with low-risk PCa, because a symptomatic man is likely to undergo prostate biopsy even if he is frail.

RS is considered the preferred framework for estimating net survival in population-based cancer studies because there is a risk of misclassifying cause of death, which is the basis for CSS (8). However, in our study both RS and CSS provided biased estimates of net survival in men with low- or intermediate-risk PCa, in particular among very old men. RS provided unrealistically high survival estimates, above 100%, whereas CSS provided unrealistically low survival estimates. Thus, the true net survival will be between the estimates of RS and CCS.

The interpretation of data on mortality in men with PCa is difficult due to old age at diagnosis, long disease trajectory, and high competing risk for death. For example, in Sweden there has been a recent increase in life expectancy, with a >40% decrease in death from myocardial infarction, leaving more older men at risk of a PCa diagnosis and PCa death (5). Hence, progress in PCa treatment might not be fully mirrored by the number of men who die of PCa. Furthermore, screening will lead to diagnoses of men with low- and intermediate-risk PCa, and despite the low biological risk of progression these men have a substantial risk of having their death incorrectly assessed as caused by PCa.

Both RS and CSS are affected by biases that drive the estimates in opposite directions. RS overestimates net survival whereas CSS underestimates it. The preferred method of net survival analysis depends on the underlying assumptions that therefore need to be evaluated considering the disease under study, characteristics of the population, and reliability of the adjudication of cause of death. The “true” net survival aims to measure the disease course in the absence of competing risk and lies somewhere between the estimates obtained by RS and CSS. Therefore, RS and CSS can be used to interpret changes in mortality rates, especially in men with PCa, of whom most die of other causes. We argue that our findings are generalizable to other populations with a similar age distribution, PCa incidence, and health-care system.

With an aging population and more widespread screening in the near future in many countries, the number of older men diagnosed with low-risk PCa will increase. The importance of correctly assessing net survival in these men will therefore be essential to evaluate cancer care. For this group of men, our access to PCa-free comparators allowed us to overcome a significant amount of bias due to incomparability in relative survival. In future studies, access to a comparative cancer-free cohort can be useful to complement analysis of net survival, and further matching on characteristics might be warranted to effectively overcome residual incomparability bias.

In conclusion, net survival is an important metric for assessing cancer care and is measured by relative survival or cause-specific survival. For men with low- and intermediate-risk PCa, both relative survival and cause-specific survival provide biased estimates of net survival. Relative survival overestimates survival and cause-specific survival underestimates survival, and the difference is particularly strong among very old men. The “true” estimate of net survival for these men will be between the estimates of relative survival and cause-specific survival. For young men and in particular in men with advanced PCa, relative survival and cause-specific survival yield very similar estimates.
